# Comparative karyomorphological study of some Indian *Cymbidium* Swartz, 1799 (Cymbidieae, Orchidaceae)

**DOI:** 10.3897/CompCytogen.v6i4.3461

**Published:** 2012-12-27

**Authors:** Santosh K. Sharma, Suman Kumaria, Pramod Tandon, Rao Rama Satyawada

**Affiliations:** 1 Department of Biotechnology and Bioinformatics; 2 Centre for Advanced Studies in Botany, North-Eastern Hill University, Shillong (Meghalaya), India; 3 Department of Botany, University of Delhi, Delhi, India

**Keywords:** Orchidaceae, mitosis, karyotype, heteromorphism, symmetry

## Abstract

Understanding the genetic resources and diversity is very important for the breeding programs and improvement of several economically important orchids like *Cymbidium*. Karyomorphological studies have been carried out on seven *Cymbidium* species, *Cymbidium aloifolium* (Linnaeus, 1753), *Cymbidium devonianum* Paxton,1843, *Cymbidium elegans* Lindley, 1828, *Cymbidium iridioides* D. Don, 1825, *Cymbidium lowianum* Rchb. f.,1877, *Cymbidium tigrinum* Parish ex Hook. f., 1864, and *Cymbidium tracyanum* L. Castle,1890, most of them endangered/threatened in their natural habitat. As reported earlier, the somatic chromosome number (2n = 40) has been observed in all the seven species. Distinct inter-specific variation was recorded in the arm ratio of few homologous pairs in the complements. Symmetrical or almost symmetrical karyotypes were prevalent; however significant asymmetry was reported in *Cymbidium iridioides* and *Cymbidium tracyanum*. The significance of karyotypic variation in speciation of the genus *Cymbidium* has been discussed. This study provides useful chromosome landmarks and evidence about genome evolution, heteromorphic chromosomes based heterozygosity, basic chromosome number and ploidy level in the genus *Cymbidium*.

## Introduction

*Cymbidium*, or boat orchid, is a myriad orchid with evergreen foliage and arching sprays of delicately colored and waxy flowers, comprising of 52 evergreen species in the subtribe Cyrtopodiinae of tribe Cymbidieae (Orchidaceae). Cymbidiums are renowned for an abundance of morpho-types, with a seemingly unending array of strange and often impressive variations, and represent a highly advanced terminal line of floral evolution in the family. The genus is characterized by a broad geographical distribution encompassing tropical and subtropical Asia, South of Papua, New Guinea and Northern Australia, and exhibits a tremendous diversity in growth habits. It comprises several such representatives capable of occupying almost every conceivable ecological situation, apart from marine environments and habitats characterized by extreme cold throughout the year. Inter-generic compatibility is giving rise to hybrid groups, which are characterized by both greater size and hybrid vigor vis-à-vis their putative parental species. Therefore, characterization of genetic resources and diversity is a clue for framing meaningful breeding programs of economically important orchids like *Cymbidium* (Wang et al. 2009, Sharma et al. 2010, 2011, 2012a, b, c, d).

A number of workers from Asiatic regions especially China and Japan focused on cytogenetical aspects of several *Cymbidium* species: *Cymbidium cyperifolium* Lindly, 1833, *Cymbidium faberi* Rolfe, 1896, *Cymbidium goeringii* Rchb. f., 1852, *Cymbidium kanran* Makino, 1902, *Cymbidium longibracteatum* Y.S. Wu et S.C. Chen, 1966, *Cymbidium qiubeiense* K.M. Feng et Li, 1980and *Cymbidium serratum* Schlechter, 1919(Aoyama and Tanaka 1988, Li et al. 2002a, 2002b, 2003, Long et al. 2000), and reported extensive details on chromosome counts in somatic as well as gametic cells, presence of B-chromosomes and aneuploidy/polyploidy. Conversely, data on Indian cymbidiums mostly restrict to chromosome counts (Mehra and Yashpal 1961, Mehra and Bawa 1962, Chennaveeraiah and Jorapur 1966, Singh 1984, Mehra and Kashyap 1983, 1984a, b, c, d). Vij and Shekhar (1987) did an enormous investigation on cytogenetical aspects of Indian cymbidiums. Recently, our group reported the karyomorphological characterization of three species of Asiatic cymbidiums: *Cymbidium eburneum*, *Cymbidium hookerianum* and *Cymbidium mastersii* (Sharma et al. 2010), as well as endomitotic events in tapetal cells of some *Cymbidium* species (Sharma et al. 2012c). The unequivocal species differentiation on the base of karyological has been hampered by almost identical chromosome numbers (2n = 40), minute differences in chromosome morphology and low heteromorphism with no clear indications for morphologically distinct satellite chromosomes.

The karyomorphological details of Indian representatives of *Cymbidium* are still ambiguous, which make it difficult to correctly estimate ploidy levels vis-à-vis karyological evolution. In addition to our earlier efforts (Sharma et al. 2010), the present study focuses on seven more *Cymbidium* species, most of them are endangered/threatened in their natural habitatnamely *Cymbidium aloifolium*, *Cymbidium devonianum*, *Cymbidium elegans*, *Cymbidium iridioides*, *Cymbidium lowianum*, *Cymbidium tigrinum*, and *Cymbidium tracyanum*, found in India, are expected to provide valuable baseline genetic data of the genus *Cymbidium*.

Cytological data on the Indian orchid flora are available for relatively few genera and most of them are restricted to chromosome counts only (Arora 1960, Sharma and Chatterji 1961, Mehra and Yashpal 1961, Mehra and Bawa 1962, Chennaveeraiah and Jorapur 1966). Sharma and Chatterji (1966), from their investigations encompassing 35 species of orchids belonging to 17 genera, reported the occurrence of a wide spectrum of basic numbers within each tribe and genus of family Orchidaceae. The genus *Cymbidium* has attracted a number of biologists from time to time to study a range of genetic aspects. However, from a cytogenetical and karyological point of view, scant reports are available (Singh 1984, Vij 1985, Vij and Shekhar 1987, Sharma et al. 2010). This genus has not found favor with cytogeneticists, perhaps owing to restricted geographical distribution, rarity of the plants in nature and difficulties in maintaining them under cultivation. Thus, the present investigation is an attempt to record karyomorphological details in more precise manner with prime objective of chromosome based genetic variation analysis in seven species of *Cymbidium*.

## Methods

The young plants belonging to seven species of *Cymbidium* were collected mainly from Arunachal Pradesh, Meghalaya and Sikkim provinces of Northeastern region of India. The plants were grown in greenhouses of North-Eastern Hill University, Shillong. For each species, a minimum of five individuals belonging to more than one population were studied. Details regarding collection of root tips, staining, chromosome complement preparation and their analysis are as described than described in Sharma et al. (2010). A minimum of five chromosome plates were analyzed per individual of the species. The standard method of chromosome classification (Battaglia 1955) of median (V), submedian (L), subtelocentric (J) and telocentric (I) based on the arm ratio of 1:1, >1:1<1:3, >1:3<1:0 and 1:0 respectively, was used for comparison. The degree of symmetry was estimated as per the scheme proposed by Stebbins (1971). The karyotype asymmetry indices were calculated following Paszko (2006) method considering the parameters: (1) shortest (SC) and longest (LC) chromosome length; (2) ratio of longest to shortest chromosome (LC/SC); (3) mean long arm length (p); (4) mean of short (q) and of total chromosome length (CL); (5) mean centromeric index (CI = 100xlength of short arm/total chromosome length); and (6) coefficient of variation in terms of chromosome length (CV_CL_) and (7) centromeric index (CV_CI_). The karyotype asymmetry index (AI) defined as the product of coefficient of variations (both CV_CL_ and CV_CI_) traduces the heterogeneity of chromosome length and/or centromeric index in a studied karyotype. As higher gets the AI index so does karyotype asymmetry, and inversely.

## Results

### Chromosome complement

**Figures 1–7.**
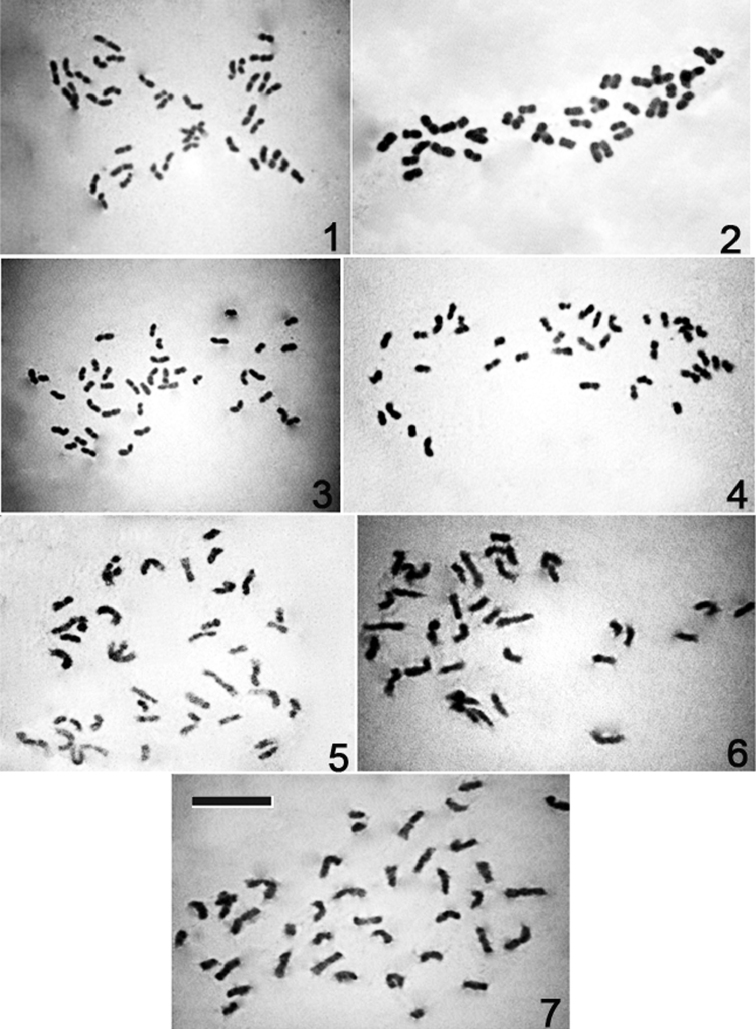
Mitotic complements of *Cymbidium* species. **1** *Cymbidium tigrinum* **2** *Cymbidium lowianum* **3** *Cymbidium devonianum* **4** *Cymbidium elegans* **5** *Cymbidium aloifolium* **6** *Cymbidium iridioides* **7** *Cymbidium tracyanum*. Bar = 10 μm.

**Figures 8–14.**
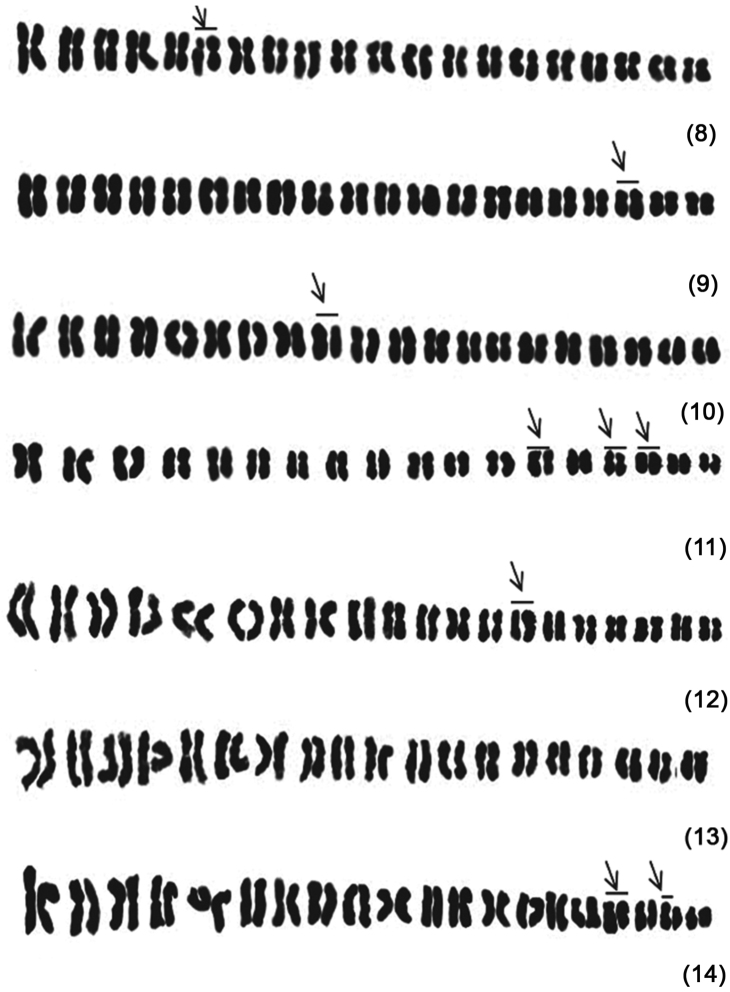
Karyotypes of *Cymbidium* species. **8** *Cymbidium tigrinum* **9** *Cymbidium lowianum* **10** *Cymbidium devonianum* **11** *Cymbidium elegans* **12** *Cymbidium aloifolium* **13** *Cymbidium iridioides* **14** *Cymbidium tracyanum*. Heteromorphic pair marked by arrows above the short arm.

The seven *Cymbidium* species presently investigated show the diploid number of 2n = 40 chromosomes in root tip cells, which were clearly resolved into 20 pairs forming a series from the longest to shortest pair within the complements. The details of karyomorphological aspects including pair-wise arm ratio, karyotypic formula, number of sub-telocentric chromosome and/or heteromorphic pairs are illustrated in Tables 1–2 and Figs 1–14. One notable feature was the lack of distinct nucleolar chromosomes in any of the seven species investigated. Variation was recorded with respect to the number of metacentric and submetacentric chromosomes, presence or absence of heteromorphic pairs in the chromosome complements ofall the seven species of *Cymbidium* (Table 1). This study revealed that the plants belonging to *Cymbidium tracyanum* are peculiar in presenting metacentric and/or submetacentric chromosomes with one pair of distinct subtelocentric chromosomes in the complement. On the other hand, all the other cymbidiums are characterized by having exclusively submetacentric and/or metacentic chromosome pairs in karyotypes and are devoid of any subtelocentrics (Table 1).The chromosome morphology with regard to a particular pair in the karyotype has shown significant variation at inter-specific level (Table 2, Figs 8–14). For example, the third pair of *Cymbidium lowianum* and *Cymbidium tracyanum* is metacentric whereas all other cymbidiums have sub-metacentric chromosomes for this particular pair. The fifth pair in *Cymbidium tracyanum*, is found to be sub-telocentric whereas, in other cymbidiums it is either metacentric or sub-metacentric. Such observation can be extended even too other pairs (i.e. IV-VIII, XIII, XVII, and XX) as well. Except for these, the rest of the pairs were found to be exclusively sub-metacentric (Table 1). Chromosome pairs VI, IX, XIV, XV, XVI, XVII, XVIII and XIX are found to be heteromorphic in *Cymbidium aloifoium*, *Cymbidium devonianum*, *Cymbidium elegans*, *Cymbidium lowianum*, *Cymbidium mastersii*, *Cymbidium tigrinum* and *Cymbidium tracyanum*, respectively. The highest number of heteromorphic pairs i.e. three (XV, XVII, and XVIII) are recorded in *Cymbidium elegans*, (Fig. 11) followed by *Cymbidium tracyanum* (Fig. 14) which had two heteromorphic pairs (XVII and XIX). Alternatively, not a single pair of the chromosome was found to be heteromorphic in *Cymbidium iridioides* (Table 1 and Fig. 13).

### Asymmetry

Following the classification of Stebbins (1971), the karyotypes of five species of *Cymbidium* (*Cymbidium aloifolium*, *Cymbidium devonianum*, *Cymbidium elegans*, *Cymbidium lowianum* and *Cymbidium tigrinum*) were resolved into 2B category while 2C and 3B types were recorded in *Cymbidium iridioides* and *Cymbidium tracyanum* , respectively (Table 2). On the other hand, asymmetry indices estimated on the basis of chromosomal statistical data (Paszko 2006) resolved the *Cymbidium* karyotypes into the range of symmetrical to lowest asymmetrical values. On the one hand, *Cymbidium devonianum* had lowest value of AI (2.26), while *Cymbidium tracyanum* showed highest asymmetry having highest AI value (5.39) (Table 2). Karyotype asymmetry also depends on both the relative variation in chromosome length (CV_CL_) and the relative variation in centromeric index (CV_CI_). *Cymbidium tracyanum* was characterized by the highest value of both CV_CL_ and CV_CI_, and then followed by *Cymbidium aloifolium* and *Cymbidium iridioides*. Remaining species of *Cymbidium* were characterized by much lower values of both CV_CL_ and CV_CI_ (Table 2).

**Table 1.** Karyomorphology and arm ratio in *Cymbidium* species.

**Taxa**	**2n**	**r-index in different chromosomes**																			
		**I**	**II**	**III**	**IV**	**V**	**VI**	**VII**	**VIII**	**IX**	**X**	**XI**	**XII**	**XIII**	**XIV**	**XV**	**XVI**	**XVII**	**XVIII**	**XIX**	**XX**
*Cymbidium tigrinum*	40	1.21 L	1.21 L	1.21 L	1.04 V	1.04 V	1.46 L	1.2 L	1.33 L	2.54 L	1.18 L	1.84 L	1.36 L	1.29 L	1.18 L	1.45 L	2.03 L	1.43 L	1.27 L	1.48 L	1.5 L
*Cymbidium lowianum*	40	1.44 L	1.12 L	1.09 V	1.23 L	1.16 L	1.14 L	1.24 L	1.6 L	1.2 L	1.29 L	1.37 L	1.14 L	1.19 L	1.57 L	1.34 L	1.12 L	1.08 V	1.38 L	2.26 L	1.06 V
*Cymbidium devonianum*	40	1.12 L	1.25 L	1.20 L	1.44 L	1.35 L	1.27 L	1.06 V	1.7 L	1.32 L	1.33 L	1.34 L	1.33 L	1.7 L	1.48 L	1.25 L	1.16 L	1.62 L	1.17 L	1.55 L	1.35 L
*Cymbidium elegans*	40	1.24 L	1.37 L	1.1 L	2.67 L	1.21 L	1.09 V	1.25 L	1.22 L	1.46 L	1.24 L	1.25 L	1.59 L	1.09 V	1.32 L	1.54 L	1.26 L	1.43 L	1.15 L	1.33 L	1.23 L
*Cymbidium aloifolium*	40	1.63 L	1.33 L	2.46 L	1.97 L	1.36 L	2.5 L	1.06 V	1.08 V	1.9 L	2.2 L	1.67 L	1.3 L	1.08 V	1.13 L	1.22 L	1.19 L	1.16 L	1.35 L	1.72 L	1.32 L
*Cymbidium iridioides*	40	1.22 L	1.29 L	1.46 L	1.44 L	1.07 V	1.11 L	1.06 V	1.52 L	1.27 L	2.27 L	1.7 L	1.02 L	1.18 L	1.31 L	1.26 L	1.28 L	1.64 L	1.15 L	2.2 L	1.25 L
*Cymbidium tracyanum*	40	1.64 L	1.2 L	1.02 V	1.08 V	3.24 J	1.05 V	1.16 L	1.61 L	1.25 L	1.8 L	1.18 L	1.06 V	1.58 L	1.67 L	2.11 L	2.3 L	2.17 L	1.47 L	1.21 L	1.23 L

Underlined values are showing heteromorphic pairs of chromosomes.

**Table 2.** Chromosome characteristics in various *Cymbidium* species.

Taxa	Range SC-LC (μm)	Ratio LC/SC	p (μm) Mean (±SD)	q (μm) Mean (±SD)	CL (μm) Mean (±SD)	CI Mean (±SD)	CV_CL_	CV_CI_	AI (Paszko 2006)	Category of symmetry (Stebbins 1971)	Karyotypic formula (Battaglia 1955)
*Cymbidium tigrinum*	2.04–4.97	2.43	1.91 (±0.343)	1.43 (±0.373)	3.35 (±0.612)	42.46 (±5.843)	18.26	13.76	2.51	2B	4V+36L
*Cymbidium lowianum*	1.76–4.44	2.52	1.82 (±0.358)	1.47 (±0.278)	3.29 (±0.584)	44.88 (±3.913)	17.75	8.71	1.54	2B	6V+34L
*Cymbidium devonianum*	1.87–3.80	2.03	1.60 (±0.311)	1.20 (±0.279)	2.81 (±0.505)	42.84 (±5.413)	17.97	12.63	2.26	2B	2V+38L
*Cymbidium elegans*	1.56–4.29	2.75	1.74 (±0.436)	1.32 (±0.331)	3.07 (±0.672)	43.39 (±5.352)	21.88	12.33	2.69	2B	4V+36L
*Cymbidium aloifolium*	1.89–4.87	2.57	1.81 (±0.607)	1.24 (±0.364)	3.05 (±0.830)	41.18 (±7.397)	27.21	17.96	4.88	2B	6V+34L
*Cymbidium iridioides*	2.62–7.25	2.76	2.46 (±0.617)	1.88 (±0.569)	4.34 (±1.073)	43.01 (±6.260)	24.72	14.55	3.59	3B	4V+36L
*Cymbidium tracyanum*	2.01–8.81	4.38	3.09 (±1.462)	2.18 (±0.782)	5.28 (±1.462)	41.21 (±8.030)	27.68	19.48	5.39	2C	8V+30L+2J

Abbreviations: (SC) shortest and (LC) longest chromosome length; (p) mean long arm length; (q) mean of short chromosome length; (CL) mean of total chromosome length; (CI) mean centromeric index; (CV_CL_) coefficient of variation in terms of chromosome length; (CV_CI_) coefficient of variation in terms of centromeric index; (AI) karyotype asymmetry index.

## Discussion

Felix and Guerra (2000) have published some excellent cytogenetical details on orchids especially on members of cymbidioid phylad. About 44 species belonging to cymbidioid genera were cytogenetically characterized and the pattern of karyological evolution within the group was reported. The chromosome variability reported by them ranges from 2n = 10 (*Psygmorchis pusilla* (Linnaeus, 1752) to 2n = 168 (*Oncidium* Swartz,1800species). They have also investigated various sub-tribes for chromosome counts and recorded variation both within and between sub-tribes, which was quite remarkable. They were of the opinion that orchids in general and cymbidioid phylad in particular have extensively benefited by the occurrence of variable base numbers followed by attainment of higher ploidy levels. From the review of published chromosome counts of *Cymbidium* and allied species from various parts of the world namely Brazil (Felix and Guerra 2000), China (Li et al. 2002a, 2002b, 2003) and Japan (Aoyama and Tanaka 1988, Aoyama 1989), it can be observed that barring few exceptions, the genus *Cymbidium* showed x = 10 as the basic number and therefore majority of the species revealed somatic chromosome number 2n = 40. The present investigation on cymbidiumsalso supports the earlier views with regard to x = 10 as true basic number of the genus *Cymbidium*. The genus *Cymbidium* is known for consistency in somatic chromosome numbers (2n = 40). However, certain deviant chromosome counts of 2n = 32, 38, 42 and 52 in species like *Cymbidium aloifolium*, *Cymbidium bicolor*, *Cymbidium eburneum*, *Cymbidium hookerianum*, *Cymbidium iridioides* and *Cymbidium tigrinum* are also reported (Vij and Shekhar 1987, Aoyama and Tanaka 1988, Aoyama 1989, Felix and Guerra 2000). Besides these unique observations on chromosome counts, they have also reported the occurrence of significant numbers of B-chromosomes in various *Cymbidium* species, whose number ranged from 1-5 in *Cymbidium lancifolium* and *Cymbidium javanicum*. The occasional occurrence of triploid cytotypes was another novel finding reported by Aoyoma and Tanaka (1988). In the present investigation, we do not come across such deviations in any of the materials investigated from north-east India. The absence of deviant chromosome numbers and overall symmetry also suggests that the diversification at inter-specific level has occurred without any significant numerical changes. However, one important point emerging out of the present study is that *Cymbidium tigrinum*, which is also considered as one of the Indian miniature cymbidiums exhibited somatic chromosome number of 2n = 40. Such observations differ from results of Vij and Shekhar (1987) who reported 2n = 38 (*x* = 19) originated through loss of one pair of chromosomes for this species.

In the present study, characteristic differences have been recorded in karyotypes at inter-specific level of the genus *Cymbidium*. In general, nine pairs out of twenty i.e. I-II, IX-X, XIV-XVI and XVIII to XIX, showed uniformity with respect to the chromosome morphology at inter-specific level while moderate to greater degree of variation was recorded in the remaining eleven pairs of the chromosome complements pattern. Such observations indicate the high degree of gene/genome stability in the genus. In general, it is predicted that orchid seeds, which are very small and light weight, can be wind-dispersed over long distances (Dressler 1993, Ackerman and Ward 1999, Chung et al. 2004), promoting genetic homogeneity among populations.

The karyotypes in most of the species investigated were found to be symmetrical according to Stebbins (1971) classification. The relative variation in chromosome length and centromeric position also provides a measure of the heterogeneity in a given karyotype. The karyotype of *Cymbidium tracyanum* was found to be most asymmetric by having 2C type of symmetry along with highest values of relative variation in chromosome length (CV_CL_), centromeric position (CV_CI_) as well as asymmetry index (AI). On the other hand, *Cymbidium lowianum* revealed least asymmetric having lowest values of CV_CL_ and CV_CI_ (17.75 and 8.71 respectively) as well as lowest AI (1.54), thereby confirming the high degree of genome stability with symmetric karyotypes. Further, the ratio of longest and shortest chromosome ranged from 2.03 in *Cymbidium devonianum* and 4.38 in *Cymbidium tracyanum*. The absence of nucleolus organizers in the chromosomes and deviant chromosome number (barring few cells in *Cymbidium aloifolium* and *Cymbidium tigrinum* ) accompanied by lack of any numerical and structural changes in chromosomes suggests a more or less stabilized genome of *Cymbidium* as evident in various species presently investigated. Most of the species (~70%) of the Orchidaceae are epiphytic (Dressler, 1993) including those of *Cymbidium*. All the available data on genetic diversity is biased towards terrestrial species and suggests that the gene flow of epiphytes could be more susceptible to environmental changes than other species due to the habitat, patchy distribution and specific pollination strategies (González-Astorga et al. 2004, Trapnell et al. 2004). The heteromorphic pairs recorded in *Cymbidium aloifolium*, *Cymbidium devonianum*, *Cymbidium elegans*, *Cymbidium lowianum*, *Cymbidium tigrinum* and *Cymbidium tracyanum* are indicative of heterogeneity and exhibit less genomic stability ultimately leading to help the species to attempt structural alterations as means of speciation. It is also opined that the chromosome re-patterning through either loss or gain of chromatin matter has also played a significant role in the evolution of the genus *Cymbidium* (Vij and Shekhar 1987). Not a single pair of nucleolus organizers has been observed in the form of a secondary constriction in any of the species investigated. However, physical localization of 45S rDNA in eight species of *Cymbidium* using fluorescent *in situ* hybridization (FISH) has confirmed the nucleolar nature of the chromosomes (Sharma et al. 2012a). *Cymbidium aloifolium*, *Cymbidium tigrinum* and *Cymbidium tracyanum* showed decondensed, dispersed, extended form of hybridization signals of rDNA as dots of fluorescence (transcriptionally active), whereas rest of the cymbidiums revealed condensed (non-active) forms, the genus hence showing a certain degree of heteromorphism in the size, intensities and appearance of rDNA signal. This phenomenon was earlier advocated by Nagl (1977) in case of *Cymbidium*, stating that it is unique among monocots having AT rich regions in genome. ITS sequence data have also determined the phylogeny of Asiatic cymbidiums with high bootstrap values and all three proposed subgenera could be distinguished clearly (Sharma et al. 2012b). Thus, it is opined that the genomic distribution pattern of 45S rDNA is very similar in most of the *Cymbidium* species, however, *Cymbidium aloifolium*, *Cymbidium tigrinum* and *Cymbidium tracyanum* did show variation and areconsistentlydistinguished from other cymbidiums both at chromosome and molecular levels (Sharma et al. 2012a, b, c, d).

## Concluding remarks

Karyotype similarities between *Cymbidium* species traduce the high degree of gene stability in the genus at inter-specific level and indicate lack of chromosome structural rearrangements during speciation in *Cymbidium*. The present investigation may also provide useful information on chromosome markers including heteromorphic chromosomes based speciation, basic chromosome number and ploidy level vis-à-vis genome evolution; which is more or less poorly known in the family Orchidaceae and especially in the genus *Cymbidium*.
